# Energy Efficient IoT Data Collection in Smart Cities Exploiting D2D Communications

**DOI:** 10.3390/s16060836

**Published:** 2016-06-08

**Authors:** Antonino Orsino, Giuseppe Araniti, Leonardo Militano, Jesus Alonso-Zarate, Antonella Molinaro, Antonio Iera

**Affiliations:** 1 DIIES Department, University Mediterranea of Reggio Calabria, Reggio Calabria 89100, Italy; antonino.orsino@unirc.it (A.O.); leonardo.militano@unirc.it (L.M.); antonella.molinaro@unirc.it (A.M.); antonio.iera@unirc.it (A.I.); 2 Centre Technològic de Telecommunications de Catalunya (CTTC), Barcelona 08860, Spain; jesus.alonso@cttc.es

**Keywords:** smart city, internet of things, LTE-A, Device-to-Device communications, power control, small data transmission

## Abstract

Fifth Generation (5G) wireless systems are expected to connect an avalanche of “smart” objects disseminated from the largest “Smart City” to the smallest “Smart Home”. In this vision, Long Term Evolution-Advanced (LTE-A) is deemed to play a fundamental role in the Internet of Things (IoT) arena providing a large coherent infrastructure and a wide wireless connectivity to the devices. However, since LTE-A was originally designed to support high data rates and large data size, novel solutions are required to enable an efficient use of radio resources to convey small data packets typically exchanged by IoT applications in “smart” environments. On the other hand, the typically high energy consumption required by cellular communications is a serious obstacle to large scale IoT deployments under cellular connectivity as in the case of Smart City scenarios. Network-assisted Device-to-Device (D2D) communications are considered as a viable solution to reduce the energy consumption for the devices. The particular approach presented in this paper consists in appointing one of the IoT smart devices as a collector of all data from a cluster of objects using D2D links, thus acting as an *aggregator* toward the eNodeB. By smartly adapting the Modulation and Coding Scheme (MCS) on the communication links, we will show it is possible to maximize the radio resource utilization as a function of the total amount of data to be sent. A further benefit that we will highlight is the possibility to reduce the transmission power when a more robust MCS is adopted. A comprehensive performance evaluation in a wide set of scenarios will testify the achievable gains in terms of energy efficiency and resource utilization in the envisaged D2D-based IoT data collection.

## 1. Introduction

The Internet of Things (IoT) holds the promise to improve our lives by providing innovative services conceived for a wide range of application domains, ranging from personal to industrial environments, and facing several societal challenges in various everyday-life human contexts [1]. The large number of heterogeneous and pervasive IoT devices continuously generating sensing data and connecting different “technological islands”, offers opportunities for applications and services only limited by the designer fantasy. In particular, this avalanche of new “smart” devices, opportunities and services is expected to provide a new radical evolution of what the current and future cities will be. The idea behind the new vision of “Smart Cities” [2] is to enable an improved wellbeing to the citizens from economic, social, and environmental perspective. This vision pairs well with the opportunities offered by the IoT paradigm where a large number of “smart” devices is able to monitor and process information from the surrounding environment. These devices typically operate through their virtual representations within a digital overlay information system that is built over the physical world. Therefore, the vision of our everyday furniture, food containers, and paper documents accessing the Internet is not a mirage anymore [3]. However, strong challenges arise when considering the current estimates of about 10 billions of IoT connected devices and between 24 billions and 50 billions total connections expected within the next five years [4,5]. In particular, a key concern for “Smart Cities” is the need of effective solutions to process the huge amount of information coming from their infrastructure (e.g., roads, bridges, rail/subways, airports, buildings) to then efficiently provide services to the city users. With respect to this, one of the biggest challenges is represented by the coexistence of different communication technologies (e.g., LTE, D2D, WiFi) that need to cooperatively interact to efficiently collect (in real time) the required data, to process them, and deliver the information of interest to the citizens [6]. To cope with this and according to the current Fifth Generation (5G) vision, the IoT infrastructure has been identified as a possible key technology able to provide data processing and management, actuation and analytics for real time information to be converted to usable knowledge. Therefore, we strongly believe that research is needed to enable energy efficient solutions for effective IoT communications and data forwarding to support the decision-making of city management and citizens and by this turn the city *smart* [7].

The capability of IoT devices to communicate and interact with each other without the human intervention is also known as Machine to Machine (M2M) communications [8] or Machine Type Communication (MTC) according to the 3rd Generation Partnership Project (3GPP) definition. In the context of this paper both IoT and MTC acronyms will be used depending on the specific context. In particular, IoT will be used when the focus is on the general capability of a device to connect to the Internet, whereas MTC is used when the focus is on the communication for the IoT devices [9,10]. A variety of short-range wireless technologies are proposed to guarantee connectivity among IoT devices, whereas the long-range connectivity is enabled by a wireless gateway which acts as a gate to the Internet. Many researchers and experts in the field share the opinion that the next-to-come fifth generation (5G) cellular systems will be a strong boost for the IoT deployment, where Long Term Evolution (LTE) is deemed to play a fundamental role. In particular, LTE-Advanced (LTE-A) [11], is the version of the 3GPP standards that completely fulfills the requirements set by ITU for IMT Advanced for 4G systems and guarantees high data rate, low latency, low cost per bit, high spectrum efficiency, and high system capacity. Moreover, it offers ubiquitous coverage, operates in licensed spectrum thus controlling interference, is supported by operator-based business models which offer contractual Service Level Agreements (SLAs) and ensures very high reliability. Nonetheless, such high performance comes at the cost of a high energy consumption at the device side, which can compromise its suitability for the IoT.

To overcome the high energy consumption limitation for LTE-A in the IoT, recent studies on power saving schemes have been performed by the standardization entity (*i.e.*, 3GPP) in the Release 12 [12]. In particular, technical specifications have been provided in order to help the operators to migrate MTC traffic from 2G to LTE networks via a new UE category (*UE Category “0”*) for low data rates and delay tolerant transmissions, setting the performance requirements to reduce complexity and power consumption [12]. Further progress will also be provided in Release 13, leading to: (i) 75% complexity reduction compared to Cat-1 UE; (ii) 10+ years battery life; and (iii) 15–20 dB coverage enhancement. Besides the standardization activities, some studies presented in the literature try to reduce the energy consumption of LTE-A for supporting MTC communications by introducing new (discontinuous) transmissions and reception modes in LTE [13].

**Figure 1:**
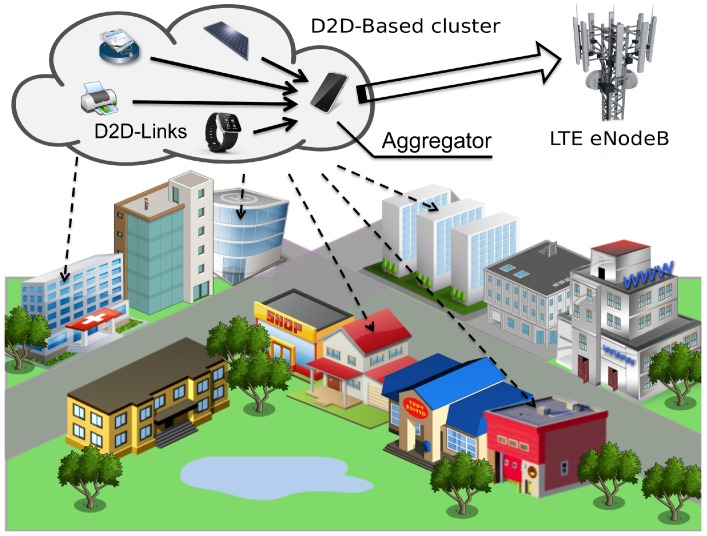
Reference scenario IoT data collection in an LTE-A cellular environment.

In this paper we follow an alternative research direction that is based on two main components: (i) the use of short-range D2D communications for energy efficient IoT data collection; and (ii) the wise use of Modulation and Coding Scheme (MCS) to convey the data in an efficient way over both the D2D and the uplink LTE-A towards the eNodeB. We focus on a typical reference IoT environment where different types of devices produce small data (in the order of few bytes) to be uploaded to the network over the LTE infrastructure, see Figure 1. Since LTE-A was originally designed for supporting data-intensive Human-Type Communications (HTC), the minimum amount of radio resources that can be allocated to a single device in LTE-A could actually be too big for the actual needs of IoT applications. Nevertheless, by smartly adapting the MCS on a communication link, it is possible to maximize the radio resource utilization and also reduce the transmission power according to the total amount of data to send [14]. This approach guarantees better energy efficiency w.r.t. classic cellular-mode uplink transmission. On the other hand, the IoT devices will be clustered together in smaller groups based on their proximity, and direct short-range D2D communications [15] are used within the cluster to improve the communication efficiency. One of the IoT smart objects in a cluster will be appointed as collector of all data from the cluster thus acting as an *aggregator* towards the eNodeB. As we will show the coupled use of D2D links [16] and wise MCS selection on all the activated links will introduce important benefits in terms energy efficiency and resource utilization.

The remainder of the paper is organized as follows. In Section 2, the background and motivations for our work are presented browsing the related work on energy efficiency in the IoT and D2D communications. In Section 3, the reference system model and service configuration are described. In Section 4, the proposed D2D cluster-based IoT data uploading scheme is presented in detail. The performance evaluation results are summarized in Section 5. The last section concludes the paper.

## 2. Background and Related Work

### 2.1. Energy Efficiency

Energy efficiency is of high interest in the context of Smart Cities and can strongly benefit from the adoption of the IoT paradigm [17] offering the opportunity to effectively manage the energy consumption of a city. For instance, city authorities and citizens might be provided with a clear overview of the amount of energy required and consumed by the different city services (e.g., public lighting, lights, surveillance camera, heating/cooling of public buildings, and so on). Moreover, the IoT can provide a useful help to identify all the entities that badly influence the city energy consumption and help the authorities to elaborate strategies to optimize their behaviors [18].

Extending the analysis to wireless networks in general, energy efficiency is of high interest in the research community to cope with the continuous growth of energy demanding applications in limited energy resource scenarios (e.g., a Smart City environment) [19]. In particular, green radio solutions are being studied for future wireless systems [20] and in [21] a discontinuous transmission mode (DTX) is introduced on the base station side to reduce the consumption of LTE communications. The discontinuous reception/transmission (DRX/DTX) mechanism in the LTE-A standard is also defined to allow devices to go in sleep mode when no data need to be received or transmitted from/to the base station. Undoubtedly, this feature is of special interest for MTCs over cellular infrastructures. In particular, in [22], the DRX/DTX optimization is investigated to maximize the sleep periods of devices while guaranteeing their Quality of Service (QoS).

Other solutions for energy efficient communication over the IoT have also been proposed in the literature e.g., based on the time reversal technique [23], whereby energy of all multi-path signals at the receiver can be harvested. Further inspiration for energy efficient solutions for data collection in the IoT can be obtained from other networking scenarios, like wireless sensor networks [24]. When considering the cellular environment, in [25] the impact of MTC on energy and delay performance is investigated from the point of view of the overload of the random access channel (RACH) whereas in [3,26] a contention-based LTE transmission mechanism is proposed showing an improvement in network resource consumption, device energy efficiency, and mean data access delay. In [27] a method to reduce the device battery consumption to efficiently support M2M communication is presented based on the proper configuration of discontinuous reception cycle length. The authors in [8] instead, propose a group based MTC approach for supporting a large number of MTC devices with small data transmissions and with different QoS requirements. In particular, MTC devices are grouped into a cluster according to their QoS characteristics and requirements. So doing, the LTE-Advanced station can manage radio resources on a cluster basis instead of a single MTC device, with a consequence complexity reduction. A further group-based scheme is proposed in [28] to minimize the overall energy consumption for the MTC devices. The proposal considers a group-based solution with the group head guaranteeing low energy consumption by limiting the access among the MTC devices and the eNB.

### 2.2. D2D Communications

The number of recent papers addressing D2D communications and its applications is wide [29] and ranges from those addressing mobile data traffic offloading [30,31], to those dealing with network coverage extension [32], or with content sharing in neighborhood areas [33,34]. This new enabling technology was introduced in the 3GPP Release-11 and further details have been defined as a technical work in 3GPP Release-12 under the name of Proximity Services (ProSe) [35].

A good taxonomy of D2D communications is given in [29], where a first distinction is made based on the spectrum adopted for D2D communications. This can be either cellular licensed spectrum, like for cellular communications (*i.e.*, inband communication), or unlicensed bands such as Wi-Fi (*i.e.*, outband communication). The inband solution, can be further classified in (i) underlay inband D2D mode and (ii) overlay inband D2D mode. In the former, D2D and cellular communications share the same licensed cellular spectrum, whereas in the latter, a portion of the cellular resources is dedicated to D2D communications for avoiding interference problems. The outband solution aims to eliminate the interference between D2D and cellular link, but needs extra interfaces such as Wi-Fi Direct or Bluetooth. Nevertheless, the research community is focusing on the inband LTE-Direct standardization (expected in the next Release-13 of the 3GPP), whereas WiFi-Direct is at the moment the “standard-de-facto” due to the many prototypes already available [36,37].

Browsing the research contributions on D2D communications, most of the work focuses on single-hop downlink services [15], whereas two-hop communications (like the one proposed in this paper), have been investigated in a very few recent papers. Specifically, in [38] a network-assisted multihop D2D communication is addressed with an analysis on D2D power control and mode selection. Similarly, a multihop D2D communication scheme is proposed in [39] for end-to-end connectivity in M2M communication. Cooperative D2D communications and green cellular communications are investigated in [40], where mobile terminals are grouped together into cooperative clusters and proximity-based transmissions are used to share content of common interest in an LTE network. In [41] instead, D2D communications are addressed in the view of adopting a cooperative approach for energy efficiency management in LTE-A public safety networks. Finally, the role that D2D communications can play in the IoT is presented in [42]. In [43] instead, the ability for devices to communicate and collaborate with each other autonomously, without any centralized control, is underlined by focusing on how state-of-the-art routing algorithms can achieve multihop D2D communication in the IoT. As discussed in [42], the use of D2D and its potential benefits may support data aggregation, where small data from several objects are aggregated locally before sending it to the final destination. However, such a promising solutions has received little attention in the literature so far.

## 3. Reference System Model

The reference scenario in this paper is a small-scale area belonging to a Smart City environment with a single LTE-A cell coverage where several IoT devices are deployed. A User Equipment (UE) in an LTE-A network can either communicate through the serving eNodeB (*i.e.*, cellular mode) or it can bypass the eNodeB and use direct communications over D2D links (*i.e.*, D2D mode). In this latter case, the eNodeB is in charge of the D2D session setup (e.g., bearer setup) [44], while power control and resource allocation procedures on the D2D links can be executed either in a distributed or in a centralized way [45]. In this paper, we assume a network-assisted D2D communications, where the coordination between radio interfaces is controlled by the LTE-A base station (*i.e.*, the eNodeB). In particular, the transmission mode (*i.e.*, either cellular- or D2D-mode), interference management and scheduling tasks are all managed by the eNodeB. Uplink cellular resources are allocated to D2D communications, which is a common choice in the literature [46], because it makes frequency reuse less challenging as the introduced interference is significantly lower w.r.t. the use of downlink resources.

The eNodeB is in charge to manage the spectrum by assigning the adequate number of Resource Block (RB) pairs to each scheduled UE and by selecting the MCS for each RB pair. Scheduling procedures are based on the *Channel Quality Indicator* (CQI) computed by the eNodeB based on the signal-to-interference-plus-noise ratio (SINR) feedback transmitted by a UE to the eNodeB. The CQI is associated to a maximum supported MCS as specified in [11] (see Table 1). This procedure is required every time a UE is accessing the LTE systems. To handle the variations of the radio channel conditions, the Adaptive Modulation and Coding (AMC) mechanism adjusts the transmission rate by selecting the proper MCS. The MCS parameters can be adapted at every *CQI Feedback Cycle* (CFC), which can last one or several Transmission Time Intervals (TTIs) (*i.e.*, 1 ms). Each radio resource includes two logical parts: the Transport Block (TB) carrying the Medium Access Control (MAC) header and the Service Data Unit; and the overhead consisting of redundancy bits generated by physical layer processing such as Cyclic Redundancy Check (CRC) insertion and channel coding. The TB Size (TBS) depends on the selected MCS. It is worth noting that when the largest available modulation scheme (64-QAM) is used, *i.e.*, when the channel quality if very good, the largest TBS can convey up to 712 bits of payload [14], which is well beyond the typical data size for most IoT applications, thus leading to low efficiency in the use of radio resources. The spectral efficiency expressed in bit/s/Hz for each MCS is reported in Table 1.

### 3.1. LTE Standard IoT Data Uploading

Let us consider an LTE-A eNodeB that receives data from a set of IoT devices within a single TTI. If the data to be sent to the eNodeB requires multiple TTIs, the same solution is applied in the consecutive TTIs. Data uploading in the standard *cellular mode* occurs through the activation of separate links from each UE to the eNodeB after the CQI collection procedure.

Formally, let K be the set of *K* LTE-A equipped devices, with each device having some data $d_{k}$ to upload in a TTI. Let *C* be the number of available CQI levels and let $c_{k}\in \left\{1,2,\ldots ,C\right\}$ be the CQI reported by device k∈K in the uplink. Each CQI level is associated to a given supported MCS. For a given MCS value *m*, the bits per RB that can be sent depend on the spectral efficiency for the given MCS, $b_{m}$ expressed in bit/s/Hz as reported in Table 1. Moreover, let R be the set of *R* radio resources (the RBs) that can be allocated to the UEs in K.

We assume the eNodeB implements a simple Round Robin allocation, whereby the whole set of radio resources is equally shared by all *cellular mode* UEs. Moreover, we assume that the transmission power for a device is equally distributed over the available RBs. Hence, the maximum uplink resources allocated to each UE will be $r_{k}=\left\lceil R/K\right\rceil ,\;\forall k\in K$. The maximum data rate for UE *k* is proportional to the number of allocated resources $r_{k}$ and the CQI level $c_{k}$. However, the IoT data is of small size (a few bytes) and typically, one RB per single TTI is enough to upload all the data and, as also pointed out in [14], leads to a low usage of the RB. In particular, the minimum IoT data size to be transmitted over an LTE network is 1 byte, to which the overhead from different headers (IP/UDP/TCP/RTP protocols) has to be added, in the range of 20–60 bytes. However, header compression solutions exist, such as the Routing and cOde-waveform CHannelization (ROCH) protocol, which drastically reduce the header size [47]. Therefore, for a single transmission we have to consider the following terms: (i) IoT application data 1 byte; (ii) a ROCH header, which can be either 1 (IPv4/UDP and IPv4/UDP/RTP cases), 3 (IPv6/UDP and IPv4/UDP/RTP cases) or 4 bytes long (IPv4/TCP and IPv6/TCP cases); (iii) a PDCP header that can be either 1 or 2 bytes long; (iv) an RLC header that can be either 1 or 2 bytes long; (v) a MAC header that can be either 2 or 3 bytes long. As a result the minimum amount of data to be transmitted is 6 bytes, whereas for IoT data also a minimum of a few additional metadata bytes should be considered.

The low efficient use of the radio resources has, at the same time, an impact on energy efficiency. In particular, with the classic *cellular mode* IoT data uploading, the energy consumption is intrinsically determined by the amount of data to upload and the efficiency $b_{m}$ for the MCS of each device. In general, we can define the energy efficiency as the ratio between the amount of data to upload expressed in bits, *D*, and the energy consumption, *E*, η=D/E. The energy consumption for user *k* over the LTE-A link can be computed as the product of the transmit power $P_{k}$ per single RB, the number of allocated RBs $r_{k}$ and the transmission time, *i.e.*, the TTI: $E_{k}=P_{k}\cdot r_{k}\cdot TTI$. Thus, the overall energy efficiency for the IoT data uploading from all *K* LTE-A equipped devices in *cellular mode* can be computed as:

(1) $$\begin{array}{c} \eta =\sum_{k\in K}\frac{d_{k}}{P_{k}\cdot r_{k}\cdot TTI} \end{array}$$

### 3.2. Energy Efficient IoT Data Uploading

Since most IoT applications are characterized by transmitting small amounts of data, the objective of reducing the power consumption in the uplink can be reached by adopting a more robust MCS which requires a lower transmit power for the device. A more robust MCS guarantees a smaller TBS, which is however acceptable as long as it can contain the data to upload. On the other hand, it might also happen that adopting a very robust MCS over multiple RBs is also more energy efficient. For an energy efficient *cellular mode* IoT data uploading, an optimal MCS selection has been proposed in [14] where IoT data are transmitted to the eNodeB with the lowest energy consumption is possible.

To evaluate the power savings with the optimal MCS selection, we first report the standard transmission power $P_{tx}$ formulation [48] for a generic UE in a subframe:

(2) $$P_{tx}=\min\left(P_{max},P_{0}+10\cdot log\left(r\right)+\alpha \cdot PL+\delta_{mcs}+f\left(\Delta_{i}\right)\right)$$

where $P_{max}$ is the maximum transmitted power of the UE, *r* is the number of Physical RBs (PRBs) allocated per user, $P_{0}$ is the target power in one RB as specified by the eNodeB to reliably demodulate and decode the data, *α* is the path loss compensation factor specified by the eNodeB in a [0,1] range, PL is the estimated UE Path Loss in uplink, $\delta_{mcs}$ is an MCS dependent offset which can be seen as the ratio between the target MCS and the basic MCS according to the UE feedback, and $f(\Delta_{i})$ is the closed loop correction function. In particular, according to the $\delta_{mcs}$ when using a higher/lower MCS level, the corresponding transmit power should be increased/decreased.

**Figure 2:**
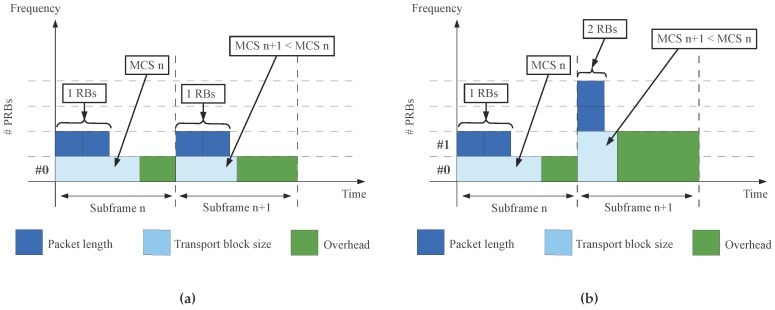
Energy efficient IoT data collection uploading solution. The overhead refers to the link layer (*i.e.*, L2) and varies according to the LTE Modulation and Coding Scheme adopted to guarantee a BLER of 10%. (**a**) Fixed number of RBs; (**b**) Variable number of RBs.

As the authors in [14] discuss, when the number of RBs is fixed to *r*, the optimal MCS to be adopted for an energy efficient solution, *i.e.*, $MCS_{r}^{*}$, is the one maximizing the TBS utilization rate:

(3) $$\begin{array}{c} MCS_{r}^{*}=\underset{MCS}{arg max}\left(\frac{D}{TBS(MCS,r)}\right)\\ s.t.D\leq TBS(MCS,r) \end{array}$$

where TBS(MCS,r) is the TBS determined by the MCS and the number of allocated resources *r*. With reference to Figure 2a, when the proposed optimization is implemented in subframe n+1 a lower MCS is being adopted w.r.t. the MCS adopted in subframe *n*. This maximization guarantees that the data is actually sent with the minimum TBS is needed, and an energy saving can be obtained due to a power decrease related to a lower MCS level. However, having a fixed value for *r* means that a UE needs to necessarily use all the allocated resources *r*. Removing this constraint, so that the number of RBs is not fixed, we can determine the most energy efficient MCS as:

(4) $$\begin{array}{c} MCS^{*}=\underset{MCS_{r}^{*}}{arg min}\left(\delta_{mcs}\left(MCS_{r}^{*}\right)\cdot r\right) \end{array}$$

where $\delta_{mcs}\left(MCS_{r}^{*}\right)$ is the power offset of each optimal MCS to the basic MCS. In such a situation, as represented in Figure 2b (see final configuration in subframe n+1 after the optimization is implemented, compared to subframe *n*), the proposed minimization allows to find the MCS with the smallest transmit power as this is equal to: $P=Pbasic\cdot \delta_{mcs}\cdot r$, where Pbasic is the power per RB for the basic MCS, $\delta_{mcs}$ is the power offset between MCS and the basic MCS.

Thus, a simple though effective solution to find the most energy efficient combination of MCS and number of RBs needed is:

- select for each RB number n=[1,r] the MCS according to Equation (3);
- from all the resulting MCS values, select the one minimizing the power transmission according to Equation (4).

In particular, in our problem setting, the value for *r* is the maximum number of allocated resources to the single device according to the radio resource allocation implemented by the eNodeB, *i.e.*, the Round Robin policy in our case. Specifically, when considering the LTE standard IoT data uploading solution illustrated in Section 3.1, the value of *r* is identically equal to $r_{k}$ (*i.e.*, the amount of RBs available for UE *k*).

## 4. The Novel D2D-Based Energy Efficient IoT Data Collection

The D2D-based solution we propose in this paper, hereafter also referred to as D2D-EE, is based on the possibility for two or more UEs to cluster together and *cooperatively upload* their data in a unique transmission to the eNodeB. In order to enhance the overall energy efficiency, we refer to an *aggregator* as the IoT device that is elected as a “cluster head” and collects all the data from the IoT devices within the cluster. Then, all the received data are aggregated into a unique payload and the aggregator itself sends them towards the LTE BS.

Since D2D links cover short-distances, the channel quality is typically good even if lower transmission power is used [49]. Consequently, short-rage communications implicitly introduce energy savings. Nonetheless, similarly to a standard uplink transmission to the eNodes, also on a D2D link further energy efficient techniques can be implemented. This means that in our scenario where IoT devices are clustered together, a more robust MCS can be used both on the cellular link from the aggregator to the eNodeB and on the D2D transmissions within the cluster. Based on this observation, the objective of the proposed energy efficient solution for the IoT data uploading is based on the following three aspects: (i) the adoption of low transmission power over short-range D2D links; (ii) an optimal energy efficient MCS selection on every D2D link within a cluster; and (iii) optimal energy efficient MCS selection in the uplink from the aggregator to the eNodeB. Specifically, for the objectives listed in (ii) and (iii), the approach presented in [14] has been extended in order to make it compliant to the specific scenario and data communication adopted in our work.

To this aim, the eNodeB will implement the algorithm described in the rest of this section. A radio resource management (RRM) scheme is implemented to configure (i) the set of UEs acting as *aggregator*; (ii) the cluster configuration for the D2D data collection; (iii) the MCS and the RBs assigned to the aggregators; and (iv) the MCS and the RBs for supporting the D2D transmissions in each cluster. In particular, when the data collection in the IoT is triggered in a single TTI, a single execution of the listed steps is executed to collect the data. Whenever significant variations in the channel conditions are observed (e.g., due to UEs’ mobility), the algorithm should be repeated to update the service configuration. However, when focusing on almost static scenarios where channel conditions and devices positions do not significantly change over time, also the formed clusters are rarely to be updated. Moreover, the additional energy consumption for the cluster formation algorithm at the LTE BS is not critical as the LTE BS is typically connected to a wired power supply system, which reduces the importance of energy consumption for the BS.

### 4.1. Assumptions for the D2D-EE Algorithm Implementation

For the proposed D2D cluster-based IoT data collection, we assume a network-assisted D2D communications where the eNodeB knows the current network state and is able to implement the proposed D2D-based IoT data collection. In particular, we consider scenarios where the IoT devices have very small computation capabilities compared to the LTE eNodeB. The eNodeB will be responsible for the allocation of the available radio resources to the cluster head(s) in the network, the so-called *aggregators* that will be in charge of uploading all IoT data from the cluster to the eNodeB. On the other hand, a completely distributed approach would require high signaling overhead for information sharing among the objects to build a shared knowledge of the network topology and the relevant channel quality information. Then, with the aim of avoiding any interference between transmissions occurring in different clusters, we assume a Round Robin radio resource allocation policy over the aggregators in the cell. In fact, this guarantees that orthogonal frequencies are used in any transmission and interference is avoided. However, enhanced radio resource management policies may be implemented for improved interference management and performance. Also for the intra-cluster D2D communications, radio resources are allocated according to a Round Robin policy, where the set of available resources is the set of resources allocated to the respective aggregator. In this way we avoid any interference within a single cluster as well. For the D2D communications we foresee a decode-and-forward (DF) relaying configuration operating in half-duplex TDD mode. First, all the data from the cluster is received by the cluster aggregator; then, the aggregator will forward all aggregated data to the eNodeB. Thus, cellular mode and D2D transmissions will never occur in the same TTI (recall that uplink resources are used for the D2D transmissions) and consequently we can assume no interference is to be managed among cellular and D2D links within a cluster.

### 4.2. Clustering for the D2D-Based Iot Data Collection

An important step for the implementation of the proposed D2D-based solution is the clustering of the IoT devices into one or multiple clusters with one aggregator per cluster. We remark that when the channel quality variations both in the uplink towards the eNodeB and among the D2D devices are significant (*i.e.*, due to mobility or channel propagation effects) the algorithm can adapt the network configuration to the environmental changes by periodically repeating the solution computation. In particular, the periodicity should be designed depending on how rapidly the environment is changing. Further, based on the cell-mode CQI values for the devices, the solution we propose for the cluster formation problem is an iterative algorithm based on the following simple steps being implemented by the eNodeB:

1. from the cell-mode CQI list sorted in descending order, select the UEs with highest cell-mode CQI levels as potential aggregators and compute for each of them the number of devices for which a D2D link is feasible;
2. out of the set of potential aggregators, the UE is selected for which the number of devices in coverage for a D2D link is the highest. Given the small data to be sent, any CQI level greater than zero on the D2D link is assumed to be sufficient to send the data;
3. the selected device will act as an aggregator and will form a D2D cluster with the devices in D2D coverage;
4. all devices belonging to the formed cluster are removed from the list; if still devices are present in the ordered list, then repeat the algorithm.

The iterative algorithm is repeated until all devices are part of a cluster or no new clusters can be formed. The output of the clustering algorithm will define the number and the size of the clusters the IoT devices, and the aggregator for each cluster. Noteworthy, the cluster size could have an influence to the communication efficiency within the cluster itself. Indeed, since a Round Robin scheduling is assumed for the radio resource allocation to the D2D transmitters in each cluster, smaller clusters mean a higher number of RBs available for each D2D transmission. As a consequence, on each D2D link the proposed energy efficient algorithm presented in Section 3.2 may introduce higher benefits. The motivation for this is that the algorithm is implemented over a larger number of RBs and has higher margins to optimize the energy and efficiency in the communication. On the contrary, if the cluster size is big, the opposite observations can be made. In particular, the extreme case is represented when only 1 RB per D2D link is available, where the benefits introduced by the algorithm are related exclusively to the use of a more robust MCS (*i.e.*, without decreasing the number of RBs).

### 4.3. The Proposed D2D-EE Solution Step by Step

**Figure 3:**
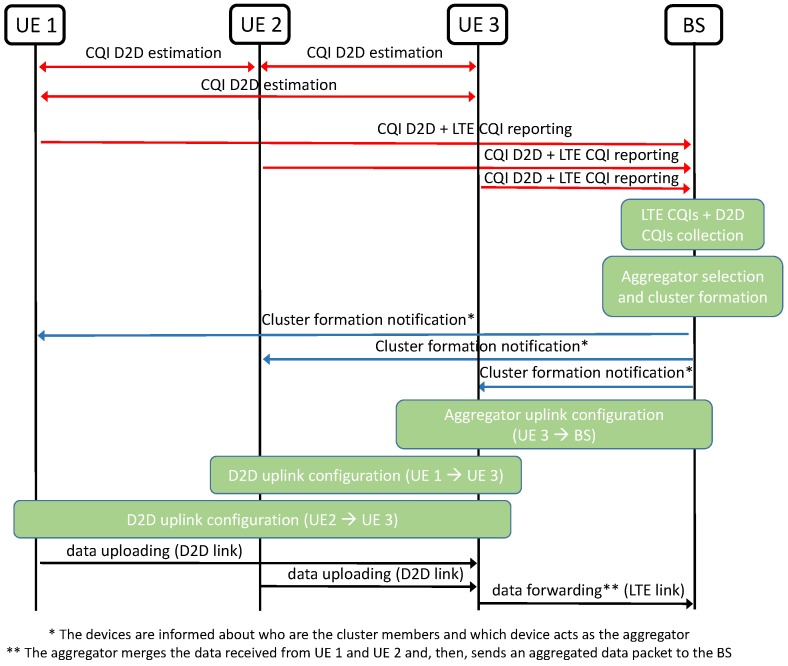
Message diagram for the proposed D2D cluster-based IoT data uploading.

The proposed D2D-EE solution foresees the implementation of the steps described below and reported in the message diagram in Figure 3.

**Cell-mode CQI collection**: The eNodeB collects the cell-mode CQI feedbacks from all IoT devices willing to upload some data, *i.e.*, $c_{k},\forall k\in K$ (note that this step is required also for any conventional solution for the devices to get access to the LTE eNodeB).

**D2D-mode CQI collection**: The eNodeB collects also the $c_{i,j}$ values from all UEs i,j∈K,i≠j; this information will be used to discover the UEs in mutual coverage for a D2D link. In particular, the eNodeB computes a D2D CQI matrix (DCM) (an example is reported in Table 2) based on the $c_{i,j}$ values for all the links between the devices (we have always $c_{i,i}=0$). Standard procedures are adopted for the CQI estimation and the rate at which the D2D channel conditions are updated can be very low [50] which induces a non-critical cost of the D2D channel quality acquisition procedure and the DCM computation. A $c_{i,j}=0$ value in the DCM indicates that a D2D link cannot be activated between devices *i* and *j*.

**Aggregator selection and cluster formation**: The information from the DCM, coupled with uplink CQI levels for all devices will be used by the eNodeB to cluster all devices in a set $S=\left\{s_{1},\ldots ,s_{S}\right\}$ of mutually disjoint clusters $s_{i}=\left\{s_{1}^{i},\ldots ,s_{|s_{i}|}^{i}\right\}$, such that $s_{i}\cap s_{i^{\prime }}=\emptyset$ for $i\neq i^{\prime }$ and $\overset{S}{\underset{i=1}{⋃}}s_{i}=K$. Let $A=\left\{a_{1},\ldots ,a_{S}\right\}$ be the set of *aggregators* in the network. The association of the devices to a cluster and the aggregator selection follows the solution described in Section 4.2. In particular, adopting a Round Robin policy for the resource allocation at the aggregators, the *mutual inter-cluster interference* is excluded.

**D2D link configuration**: For each cluster $s_{i}=\left\{s_{1}^{i},\ldots ,s_{|s_{i}|}^{i}\right\}\in S$, the eNodeB will define the resources and the MCS level to be used on the D2D links towards the aggregator. The D2D transmitter operates in half-duplex mode in which it cannot transmit and receive at the same time. In addition, all the devices performing a D2D connection have to remain active the time needed to upload their data to the aggregator. When all data from the cluster are received by the aggregator, this will forward the data to the eNodeB. The radio resources allocated to the D2D links within a cluster can follow a Round Robin policy where the available resources are the $r_{a}$ resources allocated to the respective aggregator. These resources are equally shared among the devices in a single cluster so that each D2D communication link in the cluster can use no more than $r^{d}=\lceil r_{a}/|s_{i}\left|\right\rceil$ RBs. Based on the $r^{d}$ RBs available on a single D2D link, the following energy efficient configuration will be implemented on each of the D2D links within the cluster:

- select for each RB number $n=[1,r^{d}]$ the optimal MCS, *i.e.*, $MCS_{r^{d}}^{*}$ maximizing the TBS utilization, according to Equation (5):
  (5) $$\begin{array}{c} MCS_{r^{d}}^{*}=\underset{MCS}{arg max}\left(\frac{D}{TBS(MCS,r^{d})}\right)\\ s.t.D\leq TBS(MCS,r^{d}) \end{array}$$
  where $TBS(MCS,r^{d})$ is the TBS determined by the MCS and the number of allocated resources $r^{d}$. This maximization guarantees that the data is sent with the minimum TBS is needed, and an energy saving can be obtained thanks to a power decrease related to a lower MCS level;
- from all the resulting MCS values, select the one minimizing the power transmission according to Equation (6):
  (6) $$\begin{array}{c} MCS^{*}=\underset{MCS_{r^{d}}^{*}}{arg min}\left(\delta_{mcs}\left(MCS_{r^{d}}^{*}\right)\cdot r^{d}\right) \end{array}$$
  where $\delta_{mcs}\left(MCS_{r^{d}}^{*}\right)$ is the power offset of each optimal MCS to the basic MCS.

This minimization allows to find the MCS with the smallest transmit power as this is equal to: $P^{d}=Pbasic^{d}\cdot \delta_{mcs}\cdot r^{d}$, where $Pbasic^{d}$ is the power per RB for the basic MCS on the D2D link, $\delta_{mcs}$ is the power offset between MCS and the basic MCS.

**Aggregators uplink configuration**: Once the data within a cluster have reached the aggregator, the uplink radio resources are used in cell-mode transmissions towards the eNodeB. Under the assumption of Round Robin scheduling of the radio resources, each aggregator will have no more than: $r_{a}=\left\lceil R/|S|\right\rceil$ RBs. In the uplink transmission, each aggregator will then implement the energy efficient data uploading presented in Section 3.2, to find the optimal MCS and number of RBs to adopt, where the maximum number of RBs the aggregator can use in the algorithm is exactly the $r_{a}$ value.

## 5. Performance Evaluation

A simulative analysis has been conducted by using Matlab${}^{®}$ to assess the performance of the D2D-based scheme proposed for the IoT data collection and to show its superior performance compared to the standard operation of LTE-A. In particular, we will compare three alternative solutions: (i) *LTE-A* standard solution, where the devices upload their own data through unicast link towards the LTE eNodeB; (ii) *LTE-EE* solution, where the devices implement the energy efficient solution presented in [14] on standard LTE unicast links towards the eNodeB; and (iii) *D2D-EE* solution, which is the proposed solution in this paper based on energy efficient D2D communications within clusters of devices and energy efficient unicast cellular transmissions from the cluster aggregator to the eNodeB. The key performance indicators considered in this analysis are (i) the *Transport Block utilization*; and the (ii) the *energy efficiency*.

The simulated scenario consists of an LTE eNodeB with a coverage radius equal to 250 m. In particular, the IoT devices are uniformly deployed within the LTE coverage and running the same application (e.g., smart parking, traffic congestion, smart roads, environment monitoring, weather conditions monitoring and so on). For the sake of simplicity, we assume that all the devices have to forward the same sensing data to a remote server (*i.e.*, Cloud) through the LTE eNodeB. In addition, different transmission power levels have been considered for the transmission modes used by the devices: (i) a transmitted power of 23 dBm is considered for standard LTE cell-mode uplink transmissions; whereas (ii) a power equal to 10 dBm if the devices use the D2D link [51]. Furthermore, the D2D coverage has been fixed to 50 m [49]. The focus is on a single TTI; for data requiring multiple TTIs, the same solution is applied in consecutive TTIs. Channel conditions for the UEs have been evaluated in terms of SINR experienced over each sub-carrier when path loss and fading phenomena affect the signal reception [52]. The performance analysis has been conducted by following the guidelines for the system model defined in [48] and for a number of available RBs R=100 per cell, a varying data size per device to be uploaded in the [10–100] byte range (typical values for IoT data) and a varying number of devices in the cell in the range [50–500]. In the last part of this section, also the impact of the devices density in terms of $\mathrm{devices}/{\mathrm{km}}^{2}$ within the cell is evaluated. This last analysis has the objective to show that when higher possibilities for D2D communications between devices exist, then the proposed solution performs better. For an overview of the simulation parameters please refer to Table 3.

### 5.1. Transport Block Utilization Analysis

**Figure 4:**
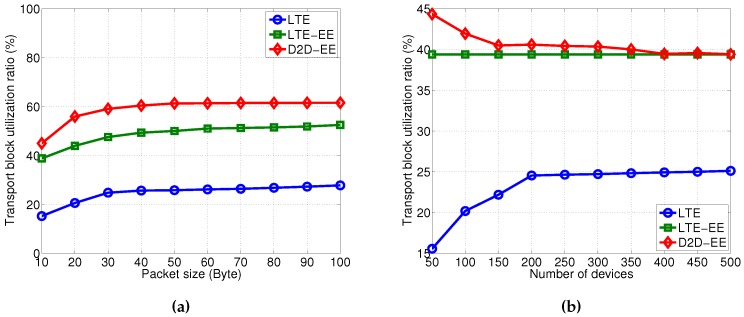
Transport Block utilization. (**a**) Varying the packet size (number of devices is 50); (**b**) Varying the number of devices (data size is 10 byte).

Let us first focus the attention on the Transport Block utilization. As we observe in Figure 4, the proposed D2D-EE always outperforms the other solutions. In particular, as expected the Transport Block utilization increases with the data size for all solutions (the number of devices is set to 50 in this case) until reaching a convergence value (around the 40 bytes value), see Figure 4a. In particular, with the D2D-EE, a maximum utilization of 62% is reached with 100 bytes to send per device, whereas the LTE-A and LTE-EE reach a maximum of 27% and 52% utilization for the same amount of bytes per device, respectively.

When, instead, we keep the packet size constant (we set it to 10 byte) and let the number of devices in the network vary, the Transport Block utilization for the D2D-EE solution decreases from a maximum of 45% utilization, to a 39% value when considering more than 400 devices. Moreover, the D2D-EE approach converges to that one of the LTE-EE when the number of devices per TTI is greater than 400. The LTE-EE, instead, shows a constant utilization percentage, 39%, independently of the number of devices, whereas the LTE-A reaches a maximum Transport Block utilization of 25% with 200 devices, starting from about 15% utilization with 50 devices. These very low utilization values are due to the very small data size which causes the cell-mode transmissions to under-utilize the RB used for transmissions. Moreover, it is important to underline that both the LTE and the LTE-EE solutions are actually never serving all the devices in the single TTI. In fact, with 100 RBs per TTI, no more than 100 devices can be served.

### 5.2. Energy Efficiency Analysis

**Figure 5:**
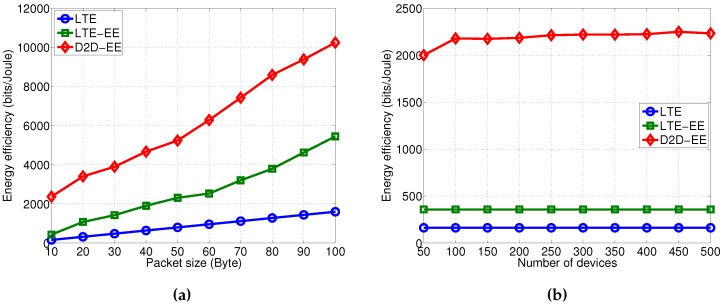
Energy efficiency. (**a**) Varying the packet size (number of devices is 50); (**b**) Varying the number of devices (data size is 10 bytes).

The second and most interesting result can be found observing the energy efficiency, expressed in bits/Joule, shown in Figure 5a. The energy efficiency increases with the packet size for all the three solutions (the number of devices is set to 50 in this case) with more emphasis for the D2D-EE and the LTE-EE solutions. In all cases the energy efficiency for the D2D-EE solution is much higher, with the highest data size (*i.e.*, 100 bytes) it is over 5 times more efficient than the LTE-A standard solution and about 2 times more efficient than the LTE-EE solution.

When considering, instead, a varying number of devices with a data size set to 10 bytes, we observe that larger number of devices make the energy efficiency increase only for the D2D-EE solution and has no impact on the LTE-A and LTE-EE solutions, see Figure 5b. At its maximum value, with 500 devices, the D2D-EE is about 6 times more efficient than the LTE-EE and about 11 times more efficient than the LTE-A solution. This very important results derive from the three contributions in the D2D-EE solution: low transmission power on D2D links, optimal energy efficient MCS selection on every D2D link within a cluster, and optimal energy efficient MCS selection in the uplink from the aggregator to the eNodeB.

### 5.3. Impact of Devices Density

**Figure 6:**
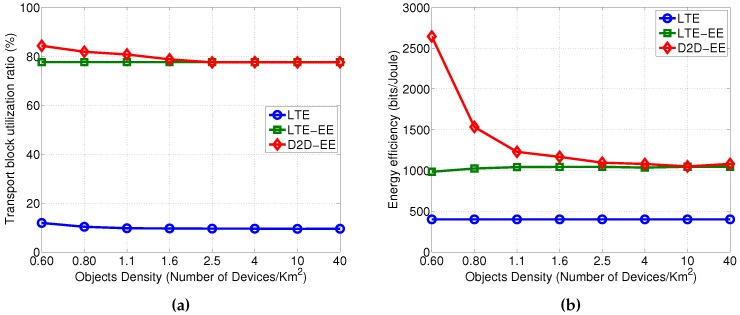
Performance for varying device density in the cell. (**a**) Transport Block utilization; (**b**) Energy efficiency.

Interesting is to understand how the distribution of the devices within the cell has an impact on the performance improvements obtained by the D2D-EE solution. In particular, the density of the devices influences the D2D communication possibilities and we will investigate up to which value of density the D2D-EE solution is still the most convenient solution. In particular, in Figure 6 the Transport Block utilization and the energy efficiency are shown for a varying value of the node density in the cell, we report the results for a [0.6–40] $\mathrm{devices}/{\mathrm{km}}^{2}$ range as this is the range where the convergence of the D2D-EE solution to the LTE-EE is visible. As we can observe from the plots, the Transport Block utilization for the D2D-EE and the LTE-EE always outperform the LTE-A solution. Moreover, the D2D-EE solution shows better performances w.r.t. the LTE-EE for values below 2.5 $\mathrm{devices}/{\mathrm{km}}^{2}$. For higher density values, that is when the devices in the cell are very densely distributed, the D2D-EE converges to the LTE-EE, as it can be seen in Figure 6a.

Also the energy efficiency for the D2D-EE converges to the LTE-EE solution for even more dense distribution of devices in the cell, *i.e.*, 10 $\mathrm{devices}/{\mathrm{km}}^{2}$, see Figure 6b. In all other cases the D2D-EE solution outperforms the LTE-EE solution from the energy efficient point of view (and the LTE-A solution as well). These results, witness to the fact that the possibility to set up D2D links among the devices is the key feature for the implementation of the D2D-EE. Nevertheless, in modern IoT scenarios the density of devices is typically high which suggests that the proposed D2D-EE solution can successfully be implemented.

### 5.4. Analysis on the Clusterization Effects

In the last part of the performance evaluation, we aim at understanding what the differences are for the performance between the aggregator and the other devices in the cluster. In particular, an aggregator has much more data to send, as it collects all data from the cluster, and at the same time it adopts a higher power in transmission over a greater number of RB pairs. We will focus the attention on a [10–100] range of devices whereas the data size varies in the [10–100] bytes interval.

**Figure 7:**
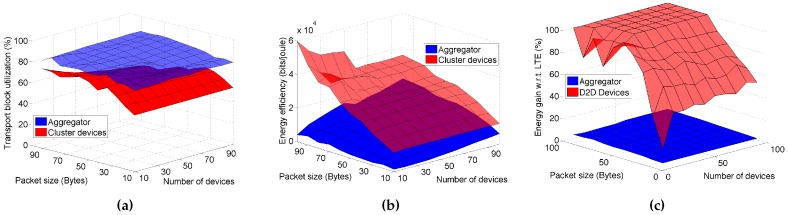
Performance for varying number of devices and data size: Aggregator *vs.* cluster devices. (**a**) Transport Block utilization; (**b**) Energy efficiency; (**c**) Energy consumption.

In Figure 7a,b, we compare the Transport Block utilization and the energy efficiency respectively for the aggregator and the other D2D objects in the cluster. We evaluate these results with a varying number of devices in the cell and a varying data size. As we can observe, the positive effect of the proposed D2D-EE solution, has a higher impact on the aggregator for what concerns the Transport block utilization (Figure 7a), whereas concerning the energy efficiency the solution has a greater positive impact on the D2D objects. In particular, the energy efficiency for the D2D objects decreases with the number of devices and for smaller data sizes. For the aggregator instead, we notice the opposite trend for the energy efficiency. In any case, the energy efficiency for the D2D objects is always higher than the one of the aggregator (see Figure 7b). This is mainly due to the lower transmission power adopted on the D2D links. Finally, in Figure 7c we are also interested in observing the energy savings for the D2D objects and the aggregator in the cluster w.r.t. the standard LTE-A data uploading. As we can observe from the plots, the D2D objects in the cluster obtain always very high savings, reaching up to 99% energy savings, whereas for the aggregator this ranges between a maximum of 6% with a small number of devices in the network and a small data size, and a minimum close to zero when many devices are involved with large data size to send per device. This shows that *also the aggregator has an energy savings* in some cases, which is mainly due to the implementation of the energy efficient solution in cell-mode uplink transmission, and anyway there is never an energy increase for them. Nevertheless, we can observe that in the worst case the energy saving for the aggregator compared to the standard LTE solution is close to zero.

**Figure 8:**
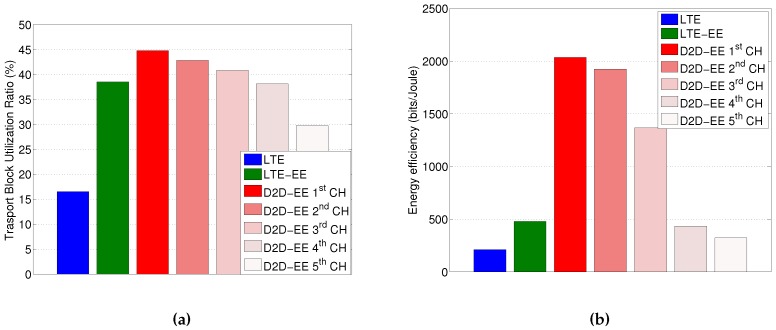
Performance with aggregator role shifting among the devices (50 devices and 10 Bytes data). (**a**) Transport Block utilization; (**b**) Energy efficiency.

To cope with this differentiation in the energy savings among the device, one could design enhanced clustering algorithms that consider an update of the configuration over time in order to share the “burden” of playing the aggregator role. Such a role-shifting approach would also mean different clusters being formed over time which may affect the efficiency of the proposed solution. For completeness in the analysis, we consider this effect in a specific study case with 50 UEs and 10 bytes of data for each device, as this is representative of the worst case when the lowest gains are obtained (clearly better performances are obtained in the other cases). The analysis is based on a policy whereby the aggregator is either a node with the best CQI level, the second best CQI level, the third best CQI level and so on, and evaluate the resulting energy efficiency and transport block utilization. What we can observe from the resulting plots reported in Figure 8, is that indeed a performance reduction is observed, but still better performance figures are obtained when choosing as cluster head the nodes until the third/fourth best CQI level. On the other hand, there are multiple devices with the same CQI level. This means that taking turns in acting as the aggregator over all the devices with the 3–4 highest CQI level towards the eNodeB, may be a good solution to share the burden and thus avoiding the cluster head to run out of its battery.

## 6. Conclusions

A novel energy efficient data collection scheme for the IoT in a Smart City scenario has been proposed, based on network-assisted D2D communications under LTE-A networks. A device, optimally selected by the eNodeB, acts as an aggregator to upload the data from the all neighboring devices, which form a cluster. All the devices in the cluster send their data to the aggregator over low power D2D links. With this approach, it is possible to overcome the low efficiency of LTE-A to handle small data transmissions. The optimal MCS is selected both on the cell-mode transmissions between the aggregator and the eNodeB and on the D2D links within a cluster to attain the maximum energy efficiency by adjusting the Transport Block Size to the total amount of data to be transmitted. The performance evaluation presented in this paper, covering a wide range of study cases, has shown that the proposed D2D-EE solution outperforms the standard LTE-A data uploading and an optimal energy efficient LTE-A data uploading scheme where D2D communication is not considered, named LTE-EE. The performance has been evaluated both in terms of radio resource utilization and of energy efficiency. Specifically, the most important results obtained with the proposed solution are that it allows a Transport Block utilization of 62% with 100 bytes to send per device and 50 devices and it is up to about 6 times more energy efficient than the LTE-EE and 11 times more efficient than the LTE-A solution, when the data size is of 10 byte and with 500 IoT devices. Noteworthy, thanks to the energy efficient solution and the lower transmission power over the D2D links, energy savings w.r.t. the standard LTE-A data uploading are obtained both for the devices in the cluster transmitting over D2D and for the aggregator node.

The study of the impact of the proposed solution on the end-to-end delay, as well as the study of the energy-delay trade-off introduced by this approach, remains part of our ongoing and future research work. In addition, we plan to investigate on the modeling of solutions with larger amounts of data to upload and scenarios where a multitude of IoT objects are disseminated in a Smart City (*i.e.*, in different position and with different multimedia applications) thus having different QoS requirements, as those posed by remote control and alarm applications. In these cases, large data to be sent would require several TTIs to be transferred to the eNodeB and the radio resource utilization should be optimized over several consecutive TTIs data frames. Another aspect for future analysis is the impact of mobility patterns for the IoT devices on the system-level performance for the signaling overhead in executing the proposed approach, for the radio resource allocation, the transmission mode selection and the power consumption.

## Figures and Tables

**Table 1:** CQI-MCS mapping for D2D and cell-mode communication links.

CQI	Modulation	Efficiency	Min. Rate	Efficiency	Min. Rate
Index	Scheme	D2D	D2D	Cellular	Cellular
		(bit/s/Hz)	(kbps)	(bit/s/Hz)	(kbps)
1	QPSK	0.1667	28.00	0.1523	25.59
2	QPSK	0.2222	37.33	0.2344	39.38
3	QPSK	0.3333	56.00	0.3770	63.34
4	QPSK	0.6667	112.00	0.6016	101.07
5	QPSK	1.0000	168.00	0.8770	147.34
6	QPSK	1.2000	201.60	1.1758	197.53
7	16-QAM	1.3333	224.00	1.4766	248.07
8	16-QAM	2.0000	336.00	1.9141	321.57
9	16-QAM	2.4000	403.20	2.4063	404.26
10	64-QAM	3.0000	504.00	2.7305	458.72
11	64-QAM	3.0000	504.00	3.3223	558.72
12	64-QAM	3.6000	604.80	3.9023	655.59
13	64-QAM	4.5000	756.00	4.5234	759.93
14	64-QAM	5.0000	840.00	5.1152	859.35
15	64-QAM	5.5000	924.00	5.5547	933.19

**Table 2:** LTE-D2D CQI Matrix.

	Device 1	Device 2	Device 3	...	Device *j*
device 1	0	$c_{1,2}$	$c_{1,3}$	...	$c_{1,j}$
device 2	$c_{2,1}$	0	$c_{2,3}$	...	$c_{2,j}$
...	...	...	...	...	...
device *k*	$c_{k,1}$	$c_{k,2}$	$c_{k,3}$	...	$c_{k,j}$

**Table 3:** Main Simulation Parameters.

Parameter	Value
Cell radius	250 m
Bandwidth	20 MHz
Frame Structure	Type 2 (TDD)
TTI	1 ms
TDD configuration	0
eNodeB Tx power	46 dBm
$P_{max}$ cell-mode Tx power	23 dBm
$P_{max}$ D2D-mode Tx power	10 dBm [51]
Noise power	-174 dBm/Hz
D2D transmission range	50 m [49]
Path loss (cell link)	128.1 + 37.6 log(d), d[km]
Path loss (D2D link, NLOS)	40 log(d) + 30 log(f) + 49, d[km], f[Hz]
Path loss (D2D link, LOS)	16.9 log(d) + 20 log (f/5) + 46.8, d[m], f[GHz]
Shadowing standard deviation	10 dB (cell mode); 12 dB (D2D mode)
Sub-carrier spacing	15 kHz
BLER target	10%
# of Runs	1500
